# MNX (Medium Duration Nutrition and Resistance-Vibration Exercise) Bed-Rest: Effect of Resistance Vibration Exercise Alone or Combined With Whey Protein Supplementation on Cardiovascular System in 21-Day Head-Down Bed Rest

**DOI:** 10.3389/fphys.2020.00812

**Published:** 2020-07-16

**Authors:** Patrick Guinet, James Patrick MacNamara, Matthieu Berry, Françoise Larcher, Marie-Pierre Bareille, Marc-Antoine Custaud, Anne Pavy-Le Traon, Benjamin D. Levine, Nastassia Navasiolava

**Affiliations:** ^1^Département d’Anesthésie Réanimation, Centre Hospitalier Universitaire de Rennes, Rennes, France; ^2^Centre Hospitalier de Fougères, Fougères, France; ^3^Institute for Exercise and Environmental Medicine, Texas Health Presbyterian Hospital, The University of Texas Southwestern Medical Center, Dallas, TX, United States; ^4^Ramsay Santé, Clinique des Cèdres, Toulouse, France; ^5^Laboratoire de Biochimie, Centre Hospitalier Universitaire d’Angers, Angers, France; ^6^Institut de Médecine et de Physiologie Spatiales (MEDES), Toulouse, France; ^7^Centre de Recherche Clinique, Centre Hospitalier Universitaire d’Angers, Angers, France; ^8^Mitovasc UMR INSERM 1083-CNRS 6015, Université d’Angers, Angers, France; ^9^Department of Neurology, French Reference Center for MSA, University Hospital of Toulouse, Toulouse, France; ^10^Institute of Cardiovascular and Metabolic Diseases INSERM U 1048, Toulouse, France

**Keywords:** countermeasures, simulated microgravity, cardiovascular deconditioning, resistance vibration exercise, whey protein supplementation, cardiac MRI, orthostatic tolerance, VO_2_max

## Abstract

Current inflight countermeasures do not completely prevent bone and cardiovascular changes induced by microgravity. High load Resistance Exercise combined with whole body Vibration (RVE) demonstrated benefits on bone and cardiovascular system during previous Head-Down Bed Rest (HDBR) studies. We examined the effectiveness of RVE alone or combined with a nutritional supplementation of Whey protein (NeX) on cardiovascular deconditioning. Eight male subjects (age 34 ± 8 years) in a crossover design completed three 21-day HDBR campaigns (Control-CON, RVE, and NeX). Pre and post HDBR Orthostatic Tolerance (OT) was evaluated by a 15-min head-up tilt test followed by increasing levels of Lower Body Negative Pressure (LBNP). Heart rate (HR), blood pressure (BP), and Sympathetic Index (ΣI) through spectral analysis were measured during OT test. Plasma Volume (PV), and Maximal Oxygen Uptake (VO_2_max) were measured before and after each campaign. Left ventricular mass, left ventricular end diastolic (LVEDV), end systolic (LVESV), stroke (SV) volumes, and circumferential deformation at rest and during an orthostatic stress simulated by a 30 mmHg LBNP were measured by cardiac MRI. RVE failed to prevent any change in these variables and NeX did not have any additional effect over exercise alone. In the 3 groups, (1) OT time dropped similarly (bed rest *p* < 0.001), (2) HR and ΣI were increased at rest at the end of HDBR and HR increased markedly during LBNP-tilt test, with inability to increase further the ΣI, (3) PV dropped (bed rest *p* < 0.001), along with LVEDV, LVESV and SV (*p* = 0.08, *p* < 0.001, and *p* = 0.045, respectively), (4) Left ventricle mass did not change significantly, (5) Deformation of the heart assessed by global circumferential strain was preserved and early diastolic circumferential strain rate was increased during orthostatic stress at the end of HDBR, illustrating preserved systolic and diastolic function respectively, without any difference between groups. Despite the drop in PV and LV volumes, RVE and NeX tended to alleviate the decrease in VO_2_max. In conclusion, RVE and NeX failed to prevent the cardiovascular deconditioning induced by a 21 day-HDBR.

## Introduction

Head-Down Bed Rest (HDBR) accurately reproduces several of the physiological changes induced by microgravity, including many cardiovascular, muscle and bone alterations (Pavy-Le Traon et al., 2007; Hargens and Vico, 2016; Mulavara et al., 2018). HDBR permits testing potential countermeasures, among which exercise is pivotal. As spaceflight duration may increase with potential missions to the Moon and Mars, new and more effective countermeasures against detrimental adaptations will be needed. Currently, in-flight countermeasures still fail to prevent all bone changes (Rittweger et al., 2018) and cardiovascular deconditioning (Lee et al., 2015).

Cardiovascular deconditioning resulting from spaceflight and HDBR includes increased resting heart rate (HR), orthostatic intolerance (OI), and decreased maximal oxygen uptake (VO_2_max) (Bungo et al., 1985; Convertino, 1994; Pavy-Le Traon et al., 2007). Extensive data about mechanisms of OI from previous spaceflight and HDBR studies highlight its multifactorial origin. Blood volume, autonomic function, adrenergic receptor function, vascular compliance, endothelial function, cardio-vestibular interactions, cardiac mass and left ventricular (LV) volumes are all altered by spaceflight or HDBR (Pavy-Le Traon et al., 2007; Hargens and Vico, 2016). Cardiac deconditioning is evidenced by changes in LV morphology, including reduced cardiac mass, end-diastolic volume, and stroke volume (SV) (Levine et al., 1997; Perhonen et al., 2001; Mulavara et al., 2018). Furthermore, LV relaxation is slowed after bed-rest with a presumed reduction in diastolic suction which may further impair ventricular filling, especially in the upright position (Dorfman et al., 2008; Carrick-Ranson et al., 2013). Exercise training has prevented cardiac deconditioning during bed-rest but has not improved orthostatic tolerance (OT) without concurrent volume loading, emphasizing the dual contributions of plasma volume (PV) and cardiac remodeling to the cardiovascular adaptation to microgravity (Shibata and Perhonen, 2010; Hastings et al., 2012; Ploutz-Snyder et al., 2018). Thus, further understanding of cardiac response to orthostatic stress and interventions that can prevent both cardiac deconditioning and OI are needed.

The effect of high intensity resistance exercise on a vibrating plate, so-called Resistive Vibration Exercise (RVE) has been explored during 2 long-term bedrest studies (Berlin BR) (Rittweger et al., 2006; Belavy et al., 2010a). First, a 56-day horizontal BR study explored the impact of 11 RVE sessions per week, and demonstrated a beneficial effect on bone loss, changes in bone metabolism, muscle mass loss, and muscle contractile capacity (Blottner et al., 2006; Belavy et al., 2008, 2009; Armbrecht et al., 2010; Rittweger et al., 2010). Second, a 60-day HDBR compared a high load resistive exercise (RE) to RVE, and with only 3 sessions a week showed an additional effect of RVE on bone (Belavy et al., 2011) but not on muscles (Mulder et al., 2006; Belavy et al., 2010b, 2011; Miokovic et al., 2011; Gast et al., 2012).

During these bedrest studies, cardiovascular deconditioning was not assessed extensively, but interesting vascular effects were observed: RVE attenuated the diameter decrease of leg conduit arteries (1st Berlin BR) (Bleeker et al., 2005) prevented completely (carotid artery) or partially (superficial femoral artery) the increase in wall thickness (2nd Berlin BR, van Duijnhoven et al., 2010a) and abolished the marked increase in flow mediated dilation and decrease in baseline diameter of the superficial femoral artery normally associated with prolonged bed-rest (van Duijnhoven et al., 2010b). Another BR study, using a whole body vibrating device with resistive exercise of lesser intensity, did not protect OT but prevented an increase of the sympathetic index (ΣI) (reflecting the sympathovagal balance of cardiac autonomic control) and limited the decrease of the spontaneous baroreflex sensitivity (Coupé et al., 2011).

Bone, muscle and cardiovascular benefits evidenced in Berlin BR studies motivated European Space Agency’s decision to continue the study of this high intensity RVE countermeasure, reducing further the frequency of exercise to 2 sessions per week, while comparing RVE alone to a combination of RVE with a nutritional supplementation in whey protein (Nutritional + Exercise, NeX). High protein intake and essential amino acid supplementation have shown anti-catabolic effects (Phillips et al., 2012), enhanced protein synthesis in human skeletal muscle (Paddon-Jones et al., 2004; Graf et al., 2011) and, when combined with branched chain amino acids, prevented cardiac atrophy if not remodeling during bed-rest (WISE study: Dorfman et al., 2007). Nevertheless such a regimen did not maintain lower limb muscle volume and strength during a long-term bed-rest study in women (WISE study: Trappe et al., 2007; Lee et al., 2014). Furthermore, a diet high in protein led to a low-grade metabolic acidosis which altered bone metabolism after bed-rest (Zwart et al., 2005). To neutralize these acidogenic effects of whey protein, several authors combined whey protein with potassium bicarbonate supplement in bed-rest studies (Blottner et al., 2014; Bosutti et al., 2016). This combination was not effective on skeletal muscle atrophy (Blottner et al., 2014) but attenuated disuse-induced reductions in muscle fiber oxidative capacity (Bosutti et al., 2016).

The objective of our study was to provide a large exploration of the cardiovascular effects of RVE and NeX countermeasures compared to control (CON). We hypothesized that RVE and NeX would have a beneficial effect on OT, PV, VO_2_max, cardiac mass, and cardiac volumes. Furthermore, cardiac MRI was performed during lower body negative pressure (LBNP), for the first time during a HDBR study, to evaluate cardiac volumes and function during an orthostatic stress, and to determine if RVE or NeX could be an effective countermeasure.

## Materials and Methods

### Subjects

A total of twelve healthy men (age 34 ± 8 years, body mass 70 ± 8 kg, height 176 ± 6 cm, BMI 22.4 ± 1.7 kg/m^2^, mean ± SD) were included in the study.

#### Inclusion Criteria

Inclusion criteria were healthy men (according to the performed medical tests plus laboratory analysis), 20–45 years old, BMI 20–26 kg/m^2^, height 158–190 cm, no family or personal past record of acute or chronic diseases, no psychological abnormalities, fitness assessment: <35 years: 35 ml/min/kg < VO_2_max < 60 ml/min/kg; >35 years: 30 ml/min/kg < VO_2_max < 60 ml/min/kg, mobile and active (no orthopedic, musculoskeletal or cardiovascular disorders), and not under medical attendance.

#### Exclusion Criteria

Exclusion criteria were OI, cardiac rhythm abnormalities, back pain, reported hiatal hernia, thyroid dysfunction, gastro-oesophageal reflux, diabetes, renal stones, migraines, record of thrombophlebitis (personal and family history of thrombosis), claustrophobia, tobacco, drug or alcohol dependence, reported genetic muscle or bone diseases, metallic implants, reported knee problems or joint surgery, BMD: T-score ≤ −1.5, blood collections 8 weeks or less prior the study (more than 8 ml/kg), special diet (vegan, vegetarian), reported lactose intolerance, Hepatitis A, B, C, Anti – HIV1+2 antibodies, inappropriate thoracic acoustic window and any chronic disease.

This study (registered number: 2012-A00337-36) was carried out with the recommendations of the Ethics Committee (CPP Sud-Ouest Outre-Mer I). The protocol was approved by the French Health Authorities. All subjects gave written informed consent in accordance with the Declaration of Helsinki. The study was performed by the Institute for Space Medicine and Physiology (MEDES-IMPS) in Toulouse, France, and supported by the French Spatial Agency [Centre National d’Etudes Spatiales (CNES)] and European Space Agency (ESA).

### Study Design

The experiment was organized as a prospective, crossover trial with three campaigns. The campaigns were separated by wash-out periods of 3 months. Volunteers participated in all three campaigns and were assigned in a random order to “Control” (CON), “Exercise and vibration” (RVE), or “Exercise and vibration plus Nutrition” (NeX) group. For each campaign, the subjects remained at MEDES clinic for 35 days, including 7 days of ambulatory control before HDBR (B-#), 21 days of −6° HDBR (D-#), and 7 days of ambulatory recovery period (R-#). Body weight (BW), blood pressure (BP), HR, water intake and urine volume over 24 h were measured daily. Partial water balance, defined as the difference between consumed water and urine volume, was calculated. During HDBR the subjects remained in the head-down tilt position for all activities. For the meal subjects were permitted to lie on their stomach, back or side, however, they were instructed that when in side-lying the trunk and head had to remain in the head-down position, i.e., the head could not be held up with the hand or arm as one commonly does whilst reading. Video monitoring on a 24-h basis permitted further monitoring of subject adherence to the study protocol. A standard controlled diet was followed by all subjects. Individual total energy expenditure (TEE, Kcal) was calculated, based on resting metabolic rate (RMR) measured by indirect calorimetry at B-7 and B-3, and adjusted if necessary during HDBR. TEE was covered by 30–35% total fat, 40–55% carbohydrates, and proteins according to the group. TEE was calculated as followed: during HDBR: TEE = RMR × 1.1 + 10% TEE, during ambulatory control and recovery periods: TEE = RMR × 1.4 + 10% TEE. TEE was adjusted for the training groups, with an increase (in Kcal) of 2.9 × BW on exercise days.

**FIGURE 1:**
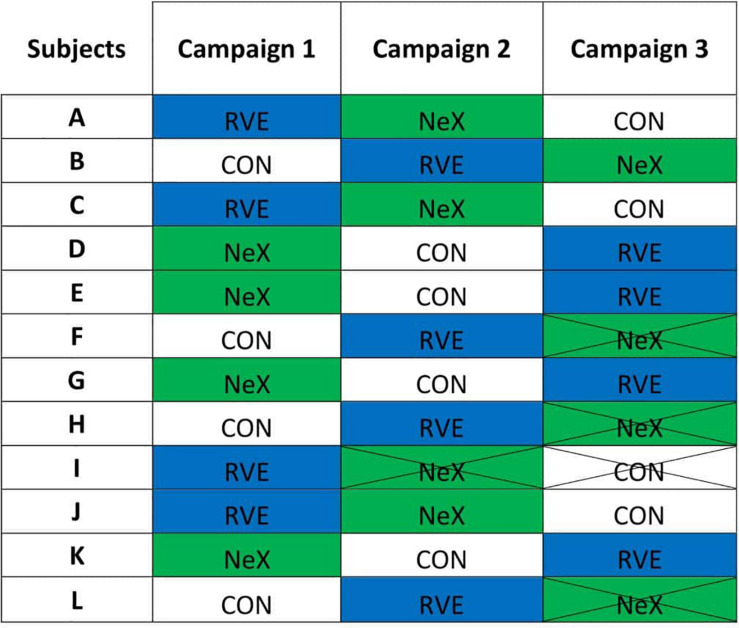
The different allocations of each subject. CON, control condition; RVE, Resistance vibration exercise condition; NeX, Resistance vibration exercise with nutritional supplementation. Campaign 2: I2 withdrawal on B-1. Campaign 3: F3 withdrawal on B-6, H3 withdrawal before the beginning of the campaign, L3 withdrawal on D-17 of HDBR.

One subject did not complete the Control protocol (withdrawal before the beginning of campaign). All subjects completed the RVE protocol. Four subjects did not complete the NeX protocol [one withdrawal before the beginning of campaign, two during ambulatory control (B-1 and B-6), and one on D-17 of HDBR]. Complete data sets were obtained in eight subjects, therefore those 8 subjects were kept for per-protocol analysis (See Figure 1).

This experiment was an integrative international study with several protocols performed on different domains. Some results on this integrative study have already been published: Cvirn et al. (2015) and Waha et al. (2015) (effects of HDBR, RVE, and NeX on the coagulation system), Kenny et al., 2017 (reduction in mitochondrial respiration partially prevented by RVE), Greaves et al., 2019 (echocardiographic study), Kermorgant et al., 2019 (cerebral autoregulation), Owen et al., 2020) (lumbar spinal muscle atrophy reduced by NeX but not by RVE alone), Graf et al., 2014 (no additional effect of whey protein supplementation compared with RVE alone regarding bone turnover markers), Pastushkova et al., 2015 (modification of urine proteome).

#### Groups and Countermeasures

##### Control (CON) group

During HDBR, volunteers did not exercise and followed a standard controlled diet. This group served to assess the effect of countermeasures.

##### Resistive vibration exercise (RVE) group

**FIGURE 2:**
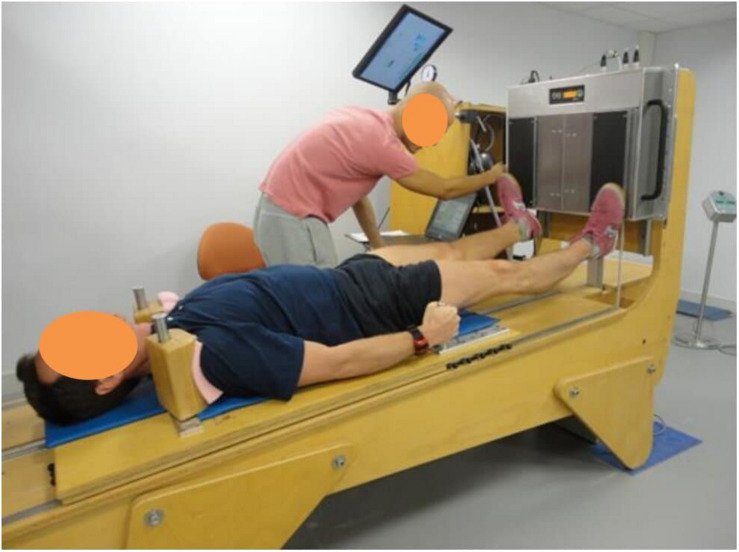
Resistive vibration exercise training (RVE and NeX groups): subjects performed all exercises in the head-down tilt position and were positioned on a moveable platform with shoulder pads and hand grips preventing downward movement and permitting application of force via the platform. A pneumatic system generated the force, applied through the moveable platform, against which the subject needed to resist and move (via the shoulder pads and hand grips). The feet were positioned on either side of a platform which was set to vibrate during high-load resistive exercises. Subjects were given visual feedback of their actual and target position in the exercise via a monitor placed in the subjects’ field of view. As the force output was dictated by the exercise device, the feedback focused on ensuring the subjects performing the exercise in the desired range of motion and at the desired speed. During the heel raises the sport scientist monitored the range of movement and encouraged the subject to go to the end of range in each direction. Here the subject is performing toe raises.

All training sessions were performed on an integrated training device, manufactured by Novotec Medical (Pforzheim, Germany), combining 2 systems already used in previous studies (Rittweger et al., 2006; Belavy et al., 2010a; Figure 2): (1) a moveable platform designed to exercise in a −6° lying position, with shoulder pads and hand grips preventing downward movement and permitting application of force generated by a pneumatic system via the platform, and (2) a vibration system: Galileo Sensor (Vibration Training device including monitoring function and data recording). The load generated by the moveable platform and transmitted by shoulder pads was progressively increased from 1.3 to 1.8 BW.

During each session, the sequence was performed as follows:

• Warm up consisted in bilateral squats from 10° to 90° knee angle during 8 s (four down, four up) controlled by metronome with eight repetitions, load: 50% of the one repetition maximum (1-RM), vibration amplitude: 8 mm, vibration frequency: 24 Hz.
• Bilateral squats from 10° to 90° knee angle during 8 s (four down, four up) controlled by metronome with 10 repetitions, load at study start: 75% of the 1-RM, progression: 5% load increase when more than 10 repetitions were possible, 5% load decrease when six or fewer repetitions were possible, vibration amplitude: 8 mm, vibration frequency: 24 Hz.
• Single leg heel raises were carried out from maximal dorsiflexion to maximal plantar flexion as fast as possible until exhaustion, 1.3 times BW, progression: 5% load increase when more than 50 s were possible, 5% load decrease when 30 s or less were possible, vibration amplitude: 8 mm, vibration frequency: 26 Hz.
• Bilateral heel raises were performed from maximal dorsiflexion to maximal plantar flexion as fast as possible until exhaustion, 1.8 times BW, progression: 5% load increase when more than 55 s were possible, 5% load decrease when 40 s or less were possible; vibration amplitude: 8 mm, vibration frequency: 26 Hz.

Each Exercise session lasted approximately 30 min. The training was performed approximately two times per week (D-2, 5, 12, 16, 21).

##### Resistive vibration exercise plus Nutrition (NeX) group

During HDBR, volunteers underwent the same physical training as previously described but received a different daily diet with an isocaloric supplementation of whey protein (0.6 g/kg BW/day). The total protein intake was 1.8 g/kg BW/day. The schedule of the protein supplementation was the following: (1) on days without exercise, supplementation was applied in equal amounts with main meal and (2) on days with exercise, half of the daily amount was taken in a timeframe of 30 min after exercise and the other half equally distributed with main meals. The product was Diaprotein^®^, a powder supplied by Nephrologische Präparate Dr. Volker Steudle (Linden, Germany). The composition was as follows: Diaprotein^®^ 100 g Powder, calories 1573 kJ (370 kcal), proteins 90 g, fat 0.2 g, lactose 2.5 g, sodium <300 mg, potassium <650 mg, calcium <400 mg, phosphorus <250 mg, and relation phosphorus/protein <3 mg/g. Since whey protein added a certain acid load to the diet, supplementation of 90 mmol potassium bicarbonate per day, applied in six portions (with main meals) was given to compensate for that. Potassium bicarbonate was provided by Krüger GmbH & Co. KG (Bergisch Gladbach, Germany). Carbohydrates and total fat were decreased in NeX subjects in order to obtain a similar caloric intake in RVE and NeX groups, and the decrease was shared between carbohydrates and fat according to the daily menu, without passing beneath 30% total fat.

### Tilt LBNP Test (Presyncopal Tilt Test)

Tilt testing with combined LBNP was chosen as the primary outcome for measuring OT. The measurement was conducted in the morning in a temperature-controlled room at baseline on B-2 and immediately following HDBR on R-0 (first rising at the end of HDBR). The subject remained supine for 20 min, after which supine data were recorded for 5 min. The tilt-table was then tilted to 80° for 15 min. After that, LBNP was applied with steps of −10 mmHg every 3 min. The test was stopped at LBNP −80 mmHg or earlier upon appearance of pre-syncopal signs, request to stop, systolic BP ≤80 mmHg, or HR<50 bpm or >170 bpm.

During the tilt-LBNP test, Orthostatic Tolerance Time (OTT, min) was measured. HR was obtained by standard electrocardiography (Biopac, ECG 100C, United States) and SBP and DBP were measured continuously with a non-invasive finger cuff method (Nexfin^®^, BMeye, United States). MBP was determined as the average value over a complete cardiac cycle. Stroke volume (SV, ml) was estimated from the Modelflow method (Wesseling et al., 1993) which computes an aortic flow waveform from finger pressure, by simulating a non-linear three-element model of the aortic input impedance. An estimate of Total Peripheral Resistance (TPR, dynes.s^–1^.cm^–5^) was calculated from Mean blood pressure (MBP)/(SV × HR). Tilt values were calculated as mean values of each variable during the last 3 available minutes of the 15-min −80° head-up tilt test.

The state of autonomic nervous system was estimated via power spectrum analysis of HR variability (HRV) (Task Force et al., 1996; Tank et al., 2004). The power spectral density was estimated using proprietary HRV software. This methodology provides the spectral markers of cardiac sympathetic [low-frequency power (LF): 0.04–0.15 Hz] and vagal [high-frequency power (HF): 0.15–0.4 Hz] modulation of the sinoatrial node activity. LF- and HF-power were determined and normalized by the total power (LF + HF). The LF-to-HF ratio (as sympathetic index, ΣI, arbitrary units – a.u), which reflects the sympathovagal balance of HR control, was calculated (Pagani et al., 1986).

Spontaneous Baroreflex Sensitivity (SBRS, ms.mmHg^–1^) was estimated using proprietary software. The assessment of baroreflex sensitivity is based on the integrative study of short-term regulation of BP and HR. A spontaneous baroreflex sequence was defined as same-direction changes in R-R interval and SBP for at least three beats. A linear regression was applied to each sequence, and the mean slope was taken as the SBRS.

### Plasma Volume Measurement

Plasma volume (PV, ml) was estimated using the optimized Carbon Monoxide Rebreathing Technique (CORT) (Schmidt and Prommer, 2005) in the morning before breakfast before HDBR on B-6 and at the end of HDBR on D-21. Hemoglobin concentration (Hb) and carbon monoxide Hb percentage were determined with a Radiometer OM 3 analyzer (Radiometer, Copenhagen, Denmark), hematocrit (Hct) with a Jouan Hematocrit A 13 centrifuge (Jouan, St. Herblain, France), and exhaled CO with a PAC 7000 analyzer (Draeger, Lübeck, Germany).

### Maximal Oxygen Uptake (VO_2_max)

An incremental dynamic leg exercise test on a cycle ergometer (Ergometrics 800S, Ergoline, Bitz, Germany) was performed at B-5 and R + 1 to determine maximal oxygen uptake (VO_2_max, ml.min^–1^.kg^–1^). Breath-by-breath VO_2_ was recorded with an Oxycon Pro metabolic cart (E. Jaeger, Hochberg, Germany). VO_2_max was determined during the subject selection period. At selection, the initial assessment of aerobic capacity was performed using the following protocol: the subjects cycled for 3 min at 30, 60, 90, 120, and 150 W, followed by an increase of 20 W every min until they could no longer maintain the desired cycling cadence of 75 rotations per minute (rpm) (peak exertion reached) and/or they wanted to stop. Before (B-5) and after the bed-rest period (R + 1), the subjects cycled for 5 min at 25, 50, and 75% of the maximal power measured at selection. At completion of the third stage, the workload increased 25 W every min until exhaustion, when the subject could no longer maintain the required cycling cadence of 75 rpm. HR and BP were monitored continuously. VO_2_max was calculated to be the highest 60-s moving average in the VO_2_ recording.

### Cardiac MRI

#### Acquisition

**FIGURE 3:**
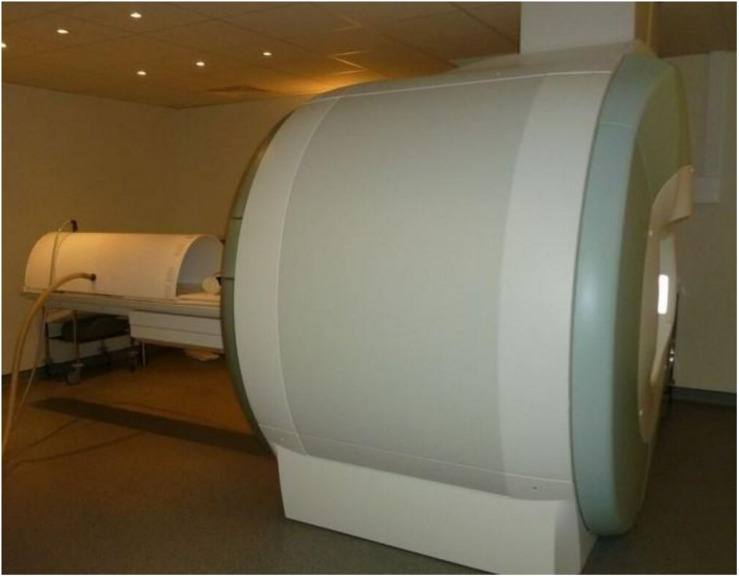
LBNP chamber, made with wood reinforced with plastic resin, especially designed for MRI scanner (Polyform^®^, La Mézière, France). Chamber was sealed with a neoprene suit. Negative pressure was maintained by air compressor (Mil’s, Lyon, France) and controlled by a manometer (ADMI, Noisy Le Grand, France) situated outside the MRI chamber.

Cardiac MRI was performed at B-3 and D-18. Subjects were transported in the supine position and placed into an MRI compatible LBNP chamber (Polyform, La Mézière, France, Figure 3). Cardiac MRI was performed on a 1.5T Philips MRI scanner during supine rest and then with LBNP. Protocol included scout and longitudinal axis images as well as short axis cine stack from ventricular apex to left atrium with slice thickness of 6 mm. After supine images without LBNP, negative pressure of −30 mmHg was maintained for 2 min prior to repeating the MRI sequence in order to simulate a mild hypovolemia approximately equivalent to the sitting position and its consequences on myocardial volumes and contractility. Negative pressure was maintained by air compressor (Mil’s, Lyon, France) and controlled by a manometer (ADMI, Noisy Le Grand, France) situated outside the MRI chamber. All MRIs and LBNP were completed under the supervision of cardiologist (MB) and anaesthesiologist (PG) at Toulouse Rangueil Hospital.

#### Analysis

Left ventricular volumes were measured by manually tracing the endocardial borders of the short axis stack of cine SSFP images from LV base to apex slices during end-diastole and end-systole using Qmass v8.1 (Medis, Raleigh, NC, United States) in accordance to CMR guidelines (Schulz-Menger et al., 2013). Papillary muscles and significant trabeculae were included in endocardial border. End-diastole was defined as the frame with the largest endocardial area before the aortic valve opened, and end-systole was defined as frame with the smallest endocardial area, after the aortic valve had closed. Care was taken to standardize the basal slice and include the LVOT in the traced blood pool. SV was determined by subtracting end-systolic volume (LVESV) from end-diastolic volume (LVEDV). Cardiac output (Q) was estimated by multiplying SV by HR during the scan. LV mass (LVM) was measured by tracing the epicardial border during end-diastole and using a semi-automated program, MassK: a threshold-based method to estimate cardiac mass (Csecs et al., 2018).

**FIGURE 4:**
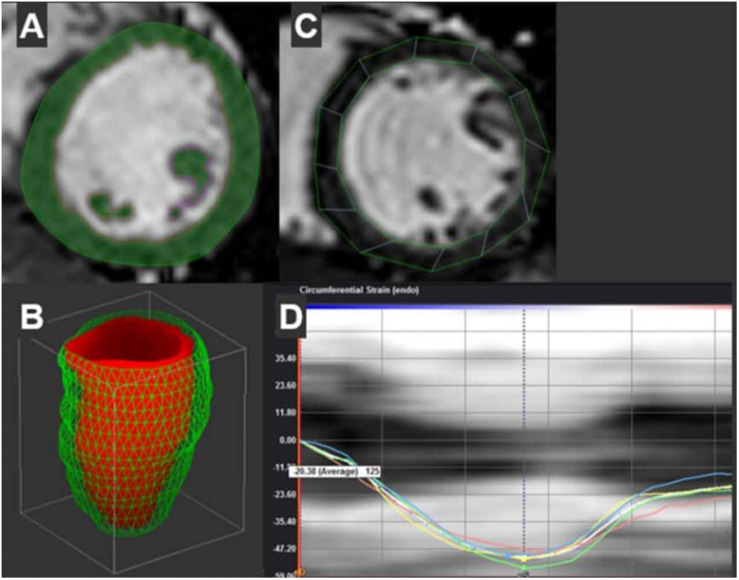
Representative contours of the left ventricle and subsequent measures are shown. **(A)** Semi-automated contours with manual adjustment are combined with Qmass’s threshold based mass detection (green shading) for each short axis slice of the ventricle. These slices create a 3D re-construction of the LV volumes by method of discs (Diastolic volume shown in Panel **B**). **(C)** Separate contours at the mid-section of the ventricle are used to measure circumferential strain through the cardiac cycle using Tomtec’s semi-automated feature tracking algorithm. **(D)** Shows both segmental strain curves. Peak strain of the mid-section is shown in white.

To assess systolic and diastolic ventricular motion, feature tracking analysis was performed using Cardiac Performance Analysis MR v1.3 (Tomtec, Unterschleissheim, Germany) (see Figure 4). The left ventricle twists during systole to generate the SV and untwists during diastole to restore the end-diastolic volume. Global circumferential strain (GCS) and early diastolic circumferential strain rate (GCSR-E) measure circumferential fiber shortening during systole and re-lengthening rate during early diastole, respectively. GCS and GCSR-E are similar to, but not interchangeable with, systolic and early diastolic torsion. Changes in GCS occur before changes in ejection fraction in some cardiovascular disease, thus GCS provides a measure of subclinical systolic and diastolic function (Lunning et al., 2015). GCS was measured using short axis stack of cine SSFP images from LV base to apex. The basal slice was identified as the slice closest to the mitral valve with circular myocardium. The apical slice was identified as the slice just proximal to systolic cavitary obliteration. The mid slice was identified between basal and apical slices with papillary muscles clearly defined (Hor et al., 2010; Claus et al., 2015). Endocardial and epicardial borders were manually traced at end-systole and adjusted at end-diastole to ensure adequate tracking. Papillary muscles and trabeculae were excluded. Feature tracking was performed by Tomtec’s semi-automated algorithm. GCS was defined as peak strain averaged from all three levels of myocardium. Early diastolic circumferential strain rate (GCSR-E) was defined as the first peak positive strain rate during diastole at the mid-section to capture the opposed untwisting of the base and apex. The observer who performed 2D and feature tracking (JM) was blinded to group assignments. Six studies were randomly selected from the study subjects to evaluate intra-observer variability for GCS and GCSR-E. The measures were repeated by researcher (JM) blinded to initial results and coefficient of variation was calculated as intra-observer variation.

### Blood Studies

Antecubital venous blood samples were collected before (B-3) and at the end of HDBR (D-21) in the morning before breakfast.

Plasma and serum samples were analyzed for electrolytes (Na+, K+, Cl−), total CO2, glucose, proteins, albumin, urea and creatinine concentrations, insulin, leptin, renin, aldosterone, brain natriuretic peptide (BNP), triglycerides, total cholesterol and HDL-cholesterol. LDL-cholesterol was calculated using the Friedewald formula. Homeostasis model assessment-insulin resistance index (HOMA-IR) was calculated as fasting insulin concentration (μU/mL) × fasting glucose concentration (mmol/L)/22.5.

### Statistical Analysis

Continuous data are expressed as mean ± SD. We first checked whether data passed d’Agostino-Pearson normality test. 3-way ANOVA or 2-way ANOVA for repeated measures were used with bed-rest (pre, post), countermeasure (Control, RVE, NeX), and position (supine, orthostatic stimulation) as within-subject factors. Statistically significant differences were further analyzed by pairwise multiple comparisons with Sidak correction. All statistical analyses were performed with GraphPrism 8.1.2. Differences were considered as statistically significant when adjusted *p* < 0.05.

## Results

### Body Weight, HR, BP, Water Balance (See Supplementary Data, Appendix 1–5)

Body weight (Supplementary Data, Appendix 1) gradually declined during HDBR without significant difference between groups (day^∗^countermeasure *p* = 0.07); at R-0 BW decrease vs. B-1 baseline was 2.3 ± 0.9 kg for CON, 2.1 ± 0.8 kg for RVE, and 1.7 ± 0.9 kg for NEX (day *p* < 0.001, countermeasure *p* = 0.66). HR (Supplementary Data, Appendix 2) expectedly increased at recovery without differences between groups (day^∗^countermeasure *p* = 0.7, day *p* < 0.001), systolic and diastolic BPs were not substantially modified (Supplementary Data, Appendix 3, 4). Expected changes in water intake and diuresis resulted to about 1L decrease in partial water balance at D-1 and about 0.8 L increase – at R-0 vs. B-1 baseline (Supplementary Data, Appendix 5).

### Presyncopal Tilt/LBNP Test

#### Orthostatic Tolerance Time (Figure 5)

Orthostatic tolerance time (min) markedly decreased in all groups without any difference between groups (bed-rest^∗^countermeasure *p* = 0.76), from 29 ± 6 to 15 ± 10 in CON, 29 ± 4 to 13 ± 9 in RVE, 27 ± 5 to 13 ± 8 in NeX (bed-rest *p* < 0.001). Pre-BR, all 8 subjects finished the 15-min upright period in all groups, post-BR there were 4 finishers in CON, 4 in RVE, 3 in NeX.

**FIGURE 5:**
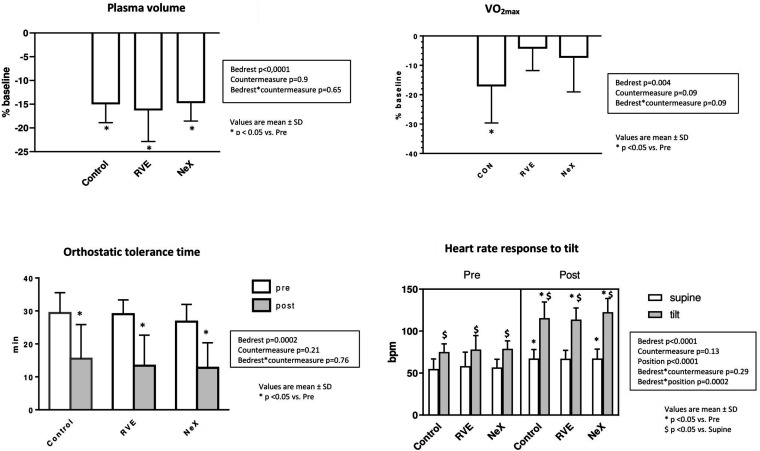
Changes in Plasma Volume (%), V02_max_(%), Orthostatic Tolerance Time (min), and Heart Rate (bpm) (supine and mean during the 3 last available minutes of the 15-min 80° head up tilt test).

#### Hemodynamic and Autonomic Responses to Tilt (Table 1 and Figure 5)

No significant differences were found in RVE and NeX compared with control group and between countermeasures (bed-rest^∗^countermeasure interaction *p* = 0.29 for HR, 0.46 for SBP, 0.57 for DBP, 0.94 for SV, 0.76 for TPR, 0.74 for ΣI, and 0.54 for SBRS, respectively).

**TABLE 1:** Presyncopal tilt/LBNP test: Hemodynamic variables at supine rest and during the 15-min 80°-head up tilt test (mean ± SD during the last 3 available minutes): Heart Rate (HR, bpm), Systolic and Diastolic Blood Pressure (SBP and DBP, mmHg), Stroke Volume (SV, ml), Total Peripheral Resistance (TPR, dynes⋅s−1⋅cm^−5^), Sympathetic Index (ΣI, a.u), i.e., ratio of low-to-high frequency spectral power, Spontaneous Baroreflex Slope (SBRS, ms⋅mmHg^−1^).

		CON		RVE		NEX		3-way ANOVA (p)				
Variable	Position	Pre-HDBR	Post-HDBR	Pre-HDBR	Post-HDBR	Pre-HDBR	Post-HDBR	BR	CM	Position (tilt)	BR *CM	BR*position
HR (bpm)	supine	55 ± 12	67 ± 11	58 ± 17	67 ± 10	57 ± 10	67 ± 11	** < 0,0001**	0,13	** < 0,0001**	0,29	**0,0002**
	tilt	75 ± 10	115 ± 9	78 ± 17	114 ± 4	79 ± 9	123 ± 16					
SBP (mmHg)	supine	130 ± 12	134 ± 9	122 ± 7	127 ± 10	124 ± 10	122 ± 9	**0,008**	**0,024**	0,25	0,46	**0,0003**
	tilt	140 ± 12	117 ± 15	128 ± 8	114 ± 11	134 ± 19	109 ± 10					
DBP (mmHg)	supine	75 ± 9	76 ± 3	70 ± 8	75 ± 5	74 ± 6	70 ± 5	0,17	0,2	** < 0,0001**	0,57	**0,046**
	tilt	89 ± 12	80 ± 8	83 ± 7	78 ± 6	87 ± 13	79 ± 6					
SV (ml)	supine	107 ± 14	102 ± 11	110 ± 15	100 ± 15	103 ± 20	104 ± 14	**0,002**	0,47	** < 0,0001**	0,94	0,098
	tilt	78 ± 15	63 ± 14	77 ± 16	66 ± 10	73 ± 16	56 ± 13					
TPR (Dynes.s^–1^cm^–5^)	supine	1390 ± 409	1164 ± 178	1212 ± 313	1178 ± 320	1333 ± 255	1081 ± 318	**0,003**	0,23	0,16	0,76	**0,006**
	tilt	1568 ± 513	1110 ± 168	1405 ± 284	1006 ± 105	1539 ± 433	1213 ± 302					
ΣI (A.U.)	supine	1,83 ± 1,34	4,04 ± 4,63	1,92 ± 1,51	3,35 ± 1,8	2,27 ± 2,86	2,53 ± 3,07	0,11	0,61	**0,02**	0,47	**0,006**
	tilt	5,74 ± 4,6	2,76 ± 1,81	6,82 ± 5,25	3,32 ± 2,55	6,14 ± 3,65	2,05 ± 1,04					
SBRS (ms.mmHg^–1^)	supine	19,6 ± 12,9	12,8 ± 5	22 ± 13,6	11,9 ± 4,7	17,3 ± 1,3	15,1 ± 6,2	**0,001**	0,7	** < 0,0001**	0,54	0,39
	tilt	8,0 ± 3,1	3,3 ± 1,8	9,0 ± 4,6	3,0 ± 1	6,6 ± 3,3	3,1 ± 1,3					

*Significant differences are bolded.*

Before HDBR, the upright position provoked expected changes in central hemodynamics and cardiac autonomic neural control (increased HR, BP, TPR, and ΣI; decreased SV and SBRS).

Post-BR supine measurements on R0 compared to pre-BR supine showed increase in HR of about 10–12 bpm, from 55 ± 12 to 67 ± 11 in CON, 58 ± 17 to 67 ± 10 in RVE, and 57 ± 10 to 67 ± 11 in NeX (bed rest *p* < 0.0001), a two-fold increase in ΣI, ∼30% decrease in SBRS, and a stable SV with the Modelflow method.

Post-BR upright measurements compared to pre-BR upright showed ∼15% decrease in SBP, ∼20% decrease in SV and more than 2-fold decrease in SBRS, accompanied by pronounced tachycardia. TPR and ΣI, which were already increased in the supine position, failed to further increase with orthostasis.

### Plasma Volume (Figure 5)

For technical reasons pre-BR PV test was not performed for the 1st campaign. Mean of pre-BR test of the 2nd and the 3rd campaign for each subject has been used as a common pre-BR baseline. RVE and NeX did not alleviate the drop in PV (interaction bed rest^∗^countermeasure *p* = 0.65). PV decreased significantly pre to post BR in the 3 groups, from 3894 ± 573 ml at baseline to 3301 ± 446 ml (−15 ± 4%) in CON, 3256 ± 519 ml (−16 ± 6%) in RVE, 3332 ± 601 ml (−15 ± 4%) in NeX, (bed rest *p* < 0.0001).

### VO_2_max (Figure 5)

The differential response to countermeasures on VO_2_max did not reach statistical significance (interaction p for bedrest^∗^countermeasure = 0.09). VO_2_max (ml/kg/min) declined significantly in CON group (from 38 ± 6 to 31 ± 5, *p* < 0.05, −17 ± 13%), but not in RVE (from 38 ± 5 to 36 ± 5, −4 ± 8%) nor in NeX groups (from 37 ± 7 to 34 ± 6, −7 ± 12%). Peak power (Watts) dropped from 266 ± 49 to 206 ± 37 in CON (−23%) and decreased from 273 ± 73 to 244 ± 46 in RVE (−10%), 248 ± 48 to 229 ± 47 in NeX (−8%).

### Cardiac Structure and Function (Figures 6, 7 and Table 2)

**FIGURE 6:**
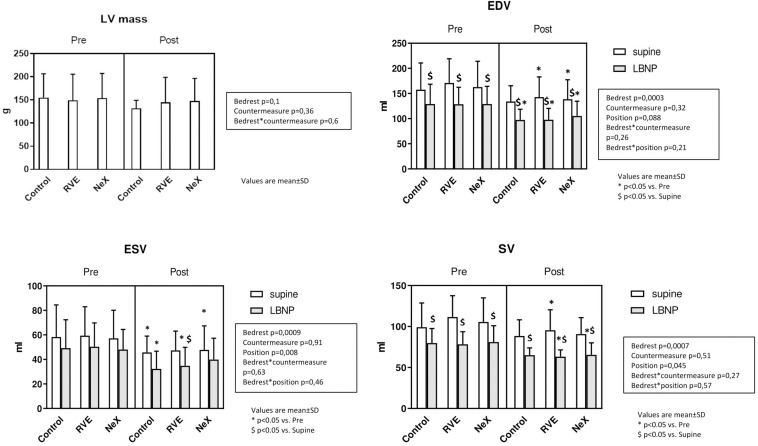
Cardiac MRI main results: Left Ventricle Mass (g), Left ventricle End-Diastolic Volume (LVEDV, ml) and End-Systolic Volume (LVESV, ml), and Stroke Volume (SV, ml).

After 18 days of HDBR, there was a small reduction in LVM that did not reach statistical significance (CON: Pre: 155 ± 52 g, end 132 ± 17 g, *p* = 0.1) (Figure 6). LVEDV decreased with LBNP prior to bedrest, at end-bedrest and with end-bedrest LBNP (LVEDV: bedrest effect *p* = 0.0003, LBNP effect *p* = 0.088) (Figure 6). LVESV similarly decreased with LBNP, end-bedrest and end-bedrest LBNP (LVESV: bedrest effect *p* = 0.0009, LBNP effect *p* = 0.008), which resulted in a decrease in SV with LBNP, end-bedrest and end-bedrest LBNP (SV LBNP effect *p* = 0.045). LV ejection fraction did not change with LBNP or bedrest. Cardiac output slightly decreased with both LBNP and bedrest (Q: bedrest effect *p* = 0.033, LBNP effect *p* = 0.005). There was no significant differential response to countermeasures on LV mass (interaction *p* = 0.6), LV volumes (LVEDV *p* = 0.26, LVESV *p* = 0.63, SV *p* = 0.57), LV ejection fraction (*p* = 0.27) or cardiac output (*p* = 0.32) (Table 2).

**TABLE 2:** Cardiac parameters pre-bedrest and at end-best with and without −30 mm Hg lower body negative pressure (LBNP).

		Control		RVE		NeX		3-way ANOVA (p)				
Variable	LBNP Level	Pre-HDBR	End-HDBR	Pre-HDBR	End-HDBR	Pre-HDBR	End-HDBR	BR	CM	Position (LBNP)	BR*CM	BR*Position
LVM (g)	0 mm Hg	155 ± 52	132 ± 17	149 ± 57	145 ± 54	154 ± 53	148 ± 49	0.1	0.36		0.6	
LVEDV (ml)	0 mm Hg	158 ± 53	134 ± 31	171 ± 48	143 ± 40	163 ± 51	139 ± 39	**0.0003**	0.32	0.088	0.26	0.21
	−30 mm Hg	129 ± 39	97 ± 21	129 ± 34	98 ± 22	129 ± 35	105 ± 29					
LVESV (ml)	0 mm Hg	58 ± 26	46 ± 13	59 ± 24	47 ± 16	57 ± 23	48 ± 20	**0.0009**	0.91	**0.008**	0.63	0.46
	−30 mm Hg	49 ± 23	32 ± 14	50 ± 19	35 ± 15	48 ± 16	40 ± 17					
SV (ml)	0 mm Hg	99 ± 29	88 ± 20	112 ± 26	95 ± 25	106 ± 29	91 ± 20	**0.0007**	0.51	**0.045**	0.27	0.57
	−30 mm Hg	80 ± 18	65 ± 9	78 ± 15	63 ± 8	81 ± 20	65 ± 15					
Ejection	0 mm Hg	0.64 ± 0.06	0.66 ± 0.04	0.66 ± 0.05	0.67 ± 0.03	0.66 ± 0.04	0.67 ± 0.06	0.32	0.97	0.32	0.27	0.63
Fraction	−30 mm Hg	0.64 ± 0.08	0.66 ± 0.04	0.66 ± 0.05	0.67 ± 0.03	0.66 ± 0.04	0.67 ± 0.06					
Cardiac Output	0 mm Hg	5.28 ± 1.2	4.97 ± 0.51	6.16 ± 1.65	5.16 ± 1.18	5.64 ± 0.68	5.1 ± 0.81	**0.033**	0.23	**0.005**	0.32	0.18
(L/min)	−30 mm Hg	4.77 ± 0.79	4.54 ± 0.46	4.86 ± 0.92	4.59 ± 0.65	4.97 ± 0.61	4.6 ± 0.67					
GCS (%)	0 mm Hg	−20.91 ± 3.58	−22.74 ± 2.53	−22.82 ± 3.67	−22.78 ± 2.48	−21.88 ± 2.72	−22.95 ± 3.31	0.34	0.85	0.06	0.41	0.21
	−30 mm Hg	−20.49 ± 3.31	−21.7 ± 3.14	−21.28 ± 3.07	−21.06 ± 3.55	−20.64 ± 2.9	−20.76 ± 3.82					
GCSR-E (1/s)	0 mm Hg	1.19 ± 0.36	1.2 ± 0.19	1.31 ± 0.3	1.23 ± 0.23	1.31 ± 0.31	1.28 ± 0.19	0.63	**0.038**	0.11	0.92	**0.02**
	−30 mm Hg	1.2 ± 0.27	1.46 ± 0.27	1.3 ± 0.21	1.5 ± 0.39	1.31 ± 0.27	1.48 ± 0.28					

*LVM, left ventricular mass; LVEDV, left ventricular end-diastolic volume; LVESV, left ventricular end-systolic volume; SV, stroke volume; GCS, global circumferential strain; GCSR-E, early diastolic circumferential strain rate. Significant differences are bolded.*

**FIGURE 7:**
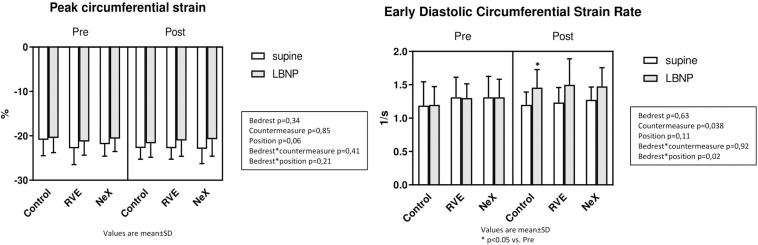
Cardiac MRI main results: Peak circumferential Strain *(%)*and Early Diastolic Circumferential Strain Rate (1/s).

Peak GCS and GCSR-E across interventions are represented in Table 2. At baseline, GCS was −20.91 ± 3.58%. Across groups, a small reduction in GCS was seen with LBNP (*p* = 0.06), but no differential change occurred after bedrest or with countermeasures (interaction for bedrest^∗^LBNP *p* = 0.21, and bedrest^∗^countermeasure *p* = 0.41) (Figure 7). GCSR-E was 1.19 ± 0.36 1/s at baseline. Across groups, GCSR-E did not change with LBNP prior to bedrest (Control pre: 1.19 ± 0.36 1/s, pre LBNP: 1.20 ± 0.27 1/s, *p* = 0.11) or after bedrest (Control pre: 1.19 ± 0.36 1/s, end: 1.20 ± 0.19 1/s, *p* = 0.63). There was a differential change with LBNP after bedrest with a significant increase in GCSR-E (Control end: 1.20 ± 0.19 1/s, end LBNP: 1.46 ± 0.27 1/s, interaction for bedrest^∗^position = 0.02) No differential effect of the countermeasures was seen (interaction *p* = 0.92). Intra-observer typical error was 1.7% for GCS and 4.8% for GCSR-E.

### Cardiovascular Hormones, Blood Variables Relevant to Metabolism, Blood Electrolytes (Table 3 and Figure 8)

Blood biochemistry remained in the physiological range. We did not observe any significant differential changes between groups.

**TABLE 3:** Blood biochemistry.

Parameters	Ref range	Control		RVE		NeX		3 way ANOVA (p)		
		Before	End	Before	End	Before	End	Bed Rest	Countermeasure	BR*CM
Aldosterone, ng/L	30-355	53 ± 42	76 ± 31	38 ± 23	106 ± 53*	44 ± 27	93 ± 54*	**0.008**	0.77	0.13
BNP, ng/L	≤ 35	16 ± 12	6 ± 2	14 ± 13	6 ± 2	12 ± 11	6 ± 2	0.06	0.38	0.07
Renin, ng/L	2.0-25.0	15 ± 17	39 ± 20*	16 ± 12	43 ± 18*	13 ± 10	38 ± 14*	**0.0001**	0.43	0.85
Leptin, ng/ml	1.2-9.5	1.49 ± 1.39	2.75 ± 3.09	2.00 ± 2.09	2.46 ± 2.37	1.71 ± 1.68	2.38 ± 2.21	0.13	0.8	0.35
Fasting glucose, mmol/L	4.1-6.1	5.0 ± 0.4	5.0 ± 0.2	4.9 ± 0.4	4.7 ± 0.3	4.8 ± 0.4	4.8 ± 0.4	0.15	0.24	0.8
Fasting insulin, μU/ml	5.0-25.0	4.3 ± 0.9	5.9 ± 1.7	4.4 ± 1.9	6.2 ± 1.9*	4.1 ± 1.6	5.2 ± 1.1	**0.005**	0.4	0.7
HOMA- IR	< 2	0.97 ± 0.22	1.29 ± 0.36	0.98 ± 0.50	1.29 ± 0.36	0.89 ± 0.38	1.11 ± 0.26	**0.005**	0.06	0.99
Proteins, g/L	65-80	64 ± 8	68 ± 4	63 ± 8	64 ± 8	64 ± 5	63 ± 6	0.33	0.73	0.21
Albumin, g/L	35-50	40 ± 2	42 ± 2	40 ± 5	40 ± 5	40 ± 2	39 ± 3	0.71	0.87	0.17
Urea, mmol/L	2.7-7.9	4.1 ± 0.4	4.9 ± 0.2*	4.1 ± 0.2	4.9 ± 0.4*	4.2 ± 0.8	6.4 ± 0.9*^#^	**0.001**	**0.005**	**0.0001**
Creatinine, μmol/L	65-105	79 ± 7	86 ± 9*	78 ± 9	83 ± 12*	76 ± 9	82 ± 8*	**0.004**	0.47	0.82
Na^+^, mmol/L	135-145	138 ± 5	141 ± 3	139 ± 5	139 ± 7	139 ± 4	136 ± 6	0.74	0.62	0.24
Cl^–^, mmol/L	95-105	102 ± 3	103 ± 2	101 ± 3	102 ± 4	102 ± 4	99 ± 5	0.37	0.34	0.13
K^+^, mmol/L	3.5-5.0	4.0 ± 0.3	3.9 ± 0.2	4.0 ± 0.4	3.9 ± 0.3	4.0 ± 0.2	4.0 ± 0.4	0.38	0.62	0.67
Total CO_2_, mmol/L	23-29	25 ± 2	26 ± 1	24 ± 3	26 ± 2	25 ± 1	26 ± 1	0.054	0.45	0.98
Cholesterol, mmol/L	3.9-5.1	4.4 ± 0.6	4.0 ± 0.6*	4.6 ± 0.7	3.8 ± 0.7*	4.8 ± 0.8^#^	3.8 ± 0.8*	**0.004**	0.97	**0.01**
HDL, mmol/L	0.9-2.3	1.2 ± 0.2	1 ± 0.2*	1.2 ± 0.3	0.9 ± 0.2*	1.2 ± 0.2	0.9 ± 0.2*	**0.001**	0.95	0.22
LDL, mmol/L	< 4.1	2.8 ± 0.3	2.6 ± 0.4	2.9 ± 0.4	2.4 ± 0.5*	3.0 ± 0.6	2.4 ± 0.6*	**0.006**	0.92	0.12
Triglycerides, mmol/L	0.3-2.3	1.10 ± 0.47	1.05 ± 0.31	1.16 ± 0.46	1.05 ± 0.41	1.28 ± 0.73	0.88 ± 0.21	0.17	0.8	0.09

Values are mean ± SD; BR*CM, bedrest*countermeasure. *p < 0.05 vs. Before; #p < 0.05 vs. Control. Significant differences are bolded.

**FIGURE 8:**
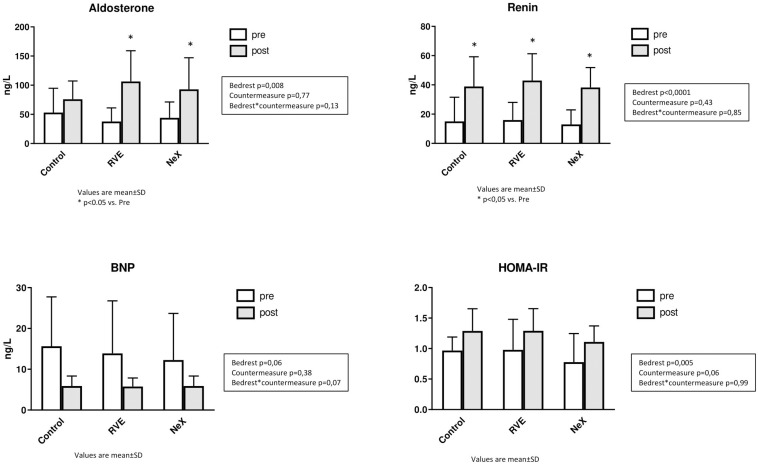
Blood biochemistry: Renin (ng.L^–1^), Aldosterone (ng.L^–1^), BNP (ng.L^–1^), and Homeostatic Model Assessment Index of Insulin Resistance (HOMA-IR).

At the end of HDBR, aldosterone and renin increased and BNP tended to decrease.

Leptin tended to increase following HDBR (*p* = 0.13). Total cholesterol decreased. HDL-fraction was unmodified. Fasting blood glucose remained stable. Fasting insulin and HOMA-IR increased.

Proteins and albumin were unchanged. Creatinine increased at the end of HDBR. Urea was also increased at the end of HDBR, with significantly more prominent increase for NeX.

Sodium, potassium and chlorine were not significantly modified by HDBR or countermeasures.

## Discussion

### The Major New Results of This Study Are the Following

(1) RVE countermeasure had no effect on HDBR-induced hypovolemia, orthostatic intolerance, modifications in heart rate and heart rate variability in responses to tilt, hormonal and metabolic changes.
(2) LV volumes (LVEDV, LVESV, SV) decreased during bedrest and orthostatic stress (related to decrease in circulating blood volume), while LV mass tended to decrease slightly. Despite reduction in size during bedrest, deformation of the heart assessed by GCS was preserved and GCSR-E was enhanced during orthostatic stress, illustrating preserved systolic and diastolic function, respectively. RVE and NeX countermeasures had no effect on cardiac structure and function.
(3) Nutritional supplementation associated with RVE did not provide any additional beneficial effect for studied variables.
(4) However, RVE and NeX countermeasures tended to limit VO_2_max loss, although there was no significant bed rest^∗^ countermeasure effect.

#### Plasma Volume, Orthostatic Tolerance

The decrease in PV observed is a well-known consequence of BR, and mimics post-spaceflight hypovolemia. Other BR studies document a decrease of 10–12% (Fortney et al., 1988, 1994; Traon et al., 1995; Custaud et al., 2002; Belin de Chantemele et al., 2004; Hastings et al., 2012).

RVE and NeX countermeasures had no effect on PV and OT. Although several previous studies had documented beneficial vascular effects of RVE during prolonged HDBR, on artery diameter, stiffness or reactivity (Bleeker et al., 2005; van Duijnhoven et al., 2010a), OT nevertheless seemed to be severely altered: at the end of the 2nd Berlin BR, 11 of 23 subjects could not finish a progressive head-up tilt (+10° every 5 min until 60° HUT) on R0 (Belavy et al., 2010b). During this 2nd Berlin BR the last exercise session was scheduled 4 days before the OT evaluation, which may be enough to limit any effect of exercise on OT (Butler et al., 1991; Khan et al., 2002). In the present study, the last exercise session was scheduled on D21, less than 18 h before the tilt/LBNP test, strengthening the conclusion that RVE had no effect on OT.

The low frequency and low duration of exercise and its focus exclusively on resistance type training likely limited the effectiveness of the countermeasures. A negative result on OT had been similarly observed with a high intensity resistive exercise on a flywheel ergometer 2–3 days per week during a longer 90-days BR (Belin de Chantemele et al., 2004). In contrast, prior studies using a greater frequency and duration have demonstrated that exercise can prevent cardiac deconditioning and maintain PV but not necessarily improve OT without concurrent volume loading (Guinet et al., 2009; Shibata and Perhonen, 2010; Hastings et al., 2012). The RVEs that our subjects performed may have failed to stimulate cardiac work or activate the muscle pump sufficiently to prevent beginning cardiac deconditioning and PV loss.

We did not observe any effect of RVE or NeX on the tilt-LBNP autonomic impairment. ΣI was increased in the supine position after HDBR, but could not increase further during tilt-LBNP test, and supine spontaneous baroreflex slope decreased, in all 3 groups with a further decrease in the HUT position after HDBR. After a 60-day HDBR, Coupé et al. (2011) reported that daily RVE prevented the increase in ΣI in standing position and alleviated the decrease in supine SBRS. While the frequencies of vibrations during exercise were close (30 Hz in the study from Coupé et al., 2011, 25 Hz in our study), there were several differences: in the study of Coupé et al. (2011): the frequency of RVE was higher (daily exercise vs. 2 sessions per week in our study), the vibration amplitude was lower (0.1 mm vs. 8 mm in our study), the intensity of exercise was much lower (low intensity resistive exercise vs. exhausting exercise in the present study), and HDBR was longer and could induce more pronounced deconditioning (60 days vs. 21 days in our study). As resistive exercise alone does not result in decreased sympathetic activity, unlike endurance training (Fagard, 2006), whole body vibration was hypothesized to act on the autonomic nervous system through plantar and muscular mechanoreceptors (Gladwell and Coote, 2002). This hypothetical effect was not observed in our study.

#### Biochemical Markers

We observed an increase in Renin and Aldosterone at the end of HDBR, without any difference between groups. Such results have been consistently described during BR studies and in flight, once the initial change in PV (taking 24 h) related to headward fluid shift is achieved (Gharib et al., 1992; Maillet et al., 1996). The decrease in PV, perceived by atrial and kidney receptors, triggers activation of Renin-Angiotensin-Aldosterone System (RAAS) and inhibition of Atrial Natriuretic Factor (ANF) (Gharib et al., 1992). These adaptations lead to a new steady state of PV, stabilized at about 15% less than preBR levels.

Brain natriuretic peptide tended to decrease in the 3 groups, presumably reflecting decreased cardiac load due to inactivity, and in line with documented cardiac atrophy. ANF and BNP share most biological properties including diuretic, natriuretic, cardiac antihypertrophic and antifibrotic properties, and vasodilation through inhibition of sympathetic nervous system and RAAS (Nishikimi et al., 2006). BNP and its more stable split-mate, NT-proBNP, released by ventricular walls in case of volumetric or barometric stimulus, are used as markers of cardiac load in clinical practice (Chen and Burnett, 2006; Ogawa and de Bold, 2012). BNP or NT-proBNP had not been previously measured during HDBR in humans. As ANF and BNP share the same triggering stimulus in the heart wall, the decrease observed in this 21-day HDBR is in line with the decrease in ANF observed in previous BR studies (Gharib et al., 1993; Belin de Chantemele et al., 2004).

Increase in insulin resistance presumably due to inactivity-related metabolic impairment is typically observed in protocols of simulated microgravity (Pavy-Le Traon et al., 2007). Increase in creatinine evidences increased muscle catabolism. As for increase in urea in NeX group, it may be related to increased protein intake. However, these metabolic changes remained in physiological range.

#### Cardiac Structure and Function

To our knowledge, this study is the first time that MRI, the gold standard for cardiac structure, has been used to assess bedrest-induced cardiac deconditioning during orthostatic stress. The subjects lost 14.8% of their cardiac mass during the control bedrest campaign, consistent with the cardiac atrophy seen in prior studies (Levine et al., 1996; Perhonen et al., 2001; Dorfman et al., 2008; Carrick-Ranson et al., 2013; Mulavara et al., 2018). The cardiac volumes, including LVEDV, LVESV, and SV, all decreased with bedrest to levels similar to pre-bedrest LBNP. This observation suggests that the upright, sitting position may well be the “regulated” position for the circulation during prolonged absence of gravitational gradients, as has been hypothesized by us and others (Norsk et al., 1995; Pancheva et al., 2006). Moreover LBNP at the end of bedrest (simulating the effect of orthostasis on cardiac morphology) amplified the reduction in cardiac volumes to levels far below the pre-bedrest baseline, consistent with a prior echocardiographic study (Carrick-Ranson et al., 2013). This finding emphasizes why orthostatic stress after bedrest results in an amplification of the reduction in SV induced by gravity, serving as the primary stimulus for post-bedrest OI.

NeX results on OT, PV, and cardiac mass and volumes were not different from RVE. While whey protein supplementation combined with resistance vibrations has prevented skeletal muscle loss in other populations (Pennings et al., 2011; Chale et al., 2013) it did not have statistical impact of cardiac mass loss in our study, though the relative drop was much less (14.8% in control arm, 3.9% in NeX arm). Thus, the impact of whey protein on cardiac mass loss, particularly if combined with more intensive exercise, may be of further interest. Addition of branched chain amino acids to the protein supplementation might also help maintaining left and right ventricle masses, as previously demonstrated during a long term bed-rest in women in the WISE study (Dorfman et al., 2007).

This study has two novel findings: first, while a small reduction in GCS was seen with LBNP, a similar reduction was not seen after bedrest. A decrease in GCS with gravitational stress has been shown before, and if cardiac adaptation to bedrest is solely due to loss of PV and cardiac preload, a similar reduction would be expected (Negishi et al., 2017). Yet, the reduction in GCS is limited to LBNP, suggesting that the actual twisting deformation of the heart is preserved despite its reduction in size. Second, cardiac untwisting has been shown to slow after bedrest, as assessed by MRI tagged analysis, but the cardiac response to orthostatic stress was unknown (Dorfman et al., 2008). In our study, we found no difference in the untwisting deformation (GCSR-E) with bedrest, but found a significant increase during orthostatic stress. This increase was likely a result of the higher HR and sympathetic activation increasing contractility (Fredholm et al., 2017). Yet the deconditioned heart was able to mount an appropriate deformation to maintain adequate, if slightly decreased, cardiac output. Both these findings would suggest the deformation of the heart adapts to preserve systolic and diastolic function during bedrest. While both torsion and circumferential strain aim to assess similar cardiac motion, the resulting measure (twisting/untwisting and deformation, respectively) are not interchangeable, and to our knowledge have not been directly compared. Thus, it remains unknown if the slowed untwisting demonstrated by Dorfman et al. (2008) and the increased deformation rate in our study are compatible findings, particularly since our finding occurred during orthostatic stress.

#### VO_2_max

The decreased VO2max in CON (−17 ± 13%) compared to pre HDBR levels is in line with previous studies of similar duration. A decrease of 1% per day until 30 days of HDBR is usually observed in absence of CM (Convertino, 1996; Watenpaugh et al., 2000), although a smaller reduction of 0.3% per day is reported by Ried-Larsen et al. in a recent meta-analysis (Ried-Larsen et al., 2017).

Interestingly the decrease in VO_2_max tended to be alleviated in RVE and NeX groups (respectively −4 ± 8% and −7 ± 12%, interaction *p* = 0.09), although the frequency and duration of training was quite low, e.g., 2 sessions per week with a total of 5 sessions along the 21 days of BR. Each exercise session lasted about 30 min, but the cumulative loading duration did not exceed 5–6 min. During the 1st Berlin BR, this high load RVE achieved lactate levels after exercise of 9–10 mmol/l, attesting to the high level of intensity (Rittweger et al., 2006). This intensity was accomplished by increasing progressively the load from 1.3 to 1.8 BW, and the frequency of vibration from 19 to 26 Hz at the end of BR. The relative protection of VO_2_max during this study suggests that this high intensity resistive exercise has additional benefit to the musculoskeletal gains seen in previous studies (Mulder et al., 2006; Armbrecht et al., 2010). Considering the marked decreases in PV and LV volumes evidenced in this study, the lower VO_2_max decrease in RVE and NeX might still represent a true finding despite the absence of a significant interaction effect, but studies with a larger sample size would be required to confirm this.

Considering those results in a whole, it is clear that the RVE countermeasure two times a week, alone or combined with whey protein supplementation, did not have any significant effect on most of the cardiovascular changes induced by 21 days of HDBR.

Regarding our cardiovascular variables, supplementation in whey protein did not show any benefit, except for a potential effect on cardiac mass as mentioned above. Conversely, some positive impact on muscle was observed before: whey protein supplementation alone alleviated disuse-induced reduction in fiber oxidative capacity during a 21-days HDBR (Bosutti et al., 2016) even if it did not attenuate lower limb muscle atrophy nor fiber type transition (Blottner et al., 2014). Furthermore, Owen et al. have recently shown in the same MNX bed rest, a reduction in paralumbar spinal atrophy with NeX countermeasure but not RVE alone (Owen et al., 2020).

Though RVE did not prevent cardiovascular deconditioning in this study, it does not preclude the interest of high intensity resistive training. Hastings et al., with a daily rowing exercise and biweekly strength training, prevented myocardial changes and maintained OT with an added oral volume load (Hastings et al., 2012). When a high intensity exercise provides a high loading impact, for example with plyometric exercises like series of jumps, noteworthy beneficial effects on bone and some aspects of the cardiovascular system are observed. During a 60-day-HDBR, a jump countermeasure consisting of 5–6 sessions per week, each session lasting 8–17 min, maintained bone mass and maximal muscle force (Kramer et al., 2017a) and also preserved resting HR and peak oxygen consumption (Kramer et al., 2017b), two major criteria of cardiovascular deconditioning. Nevertheless in this latter study the decrease in PV was not prevented (Kramer et al., 2017a), and neither OT nor cardiac mass and volumes were assessed. Integration of this jumping countermeasure with other ones especially effective on cardiac and vascular deconditioning, such as artificial gravity or LBNP, may be of further interest.

## Limitations

This study has several limitations that are worth noting: First, the sample size in our study was limited to 8 subjects who completed testing. While the crossover design allowed to reduce confounding covariates, we have “lost” 4 subjects out of 12, with unfortunately the withdrawal of 3 subjects in the NeX group during the 3rd campaign. There were potential effects of the countermeasures that did not reach conventional level of significance, namely cardiac mass and VO_2_max. Second, the duration and frequency of exercise were reduced compared to prior studies. This was done to assess the effect of twice weekly exercise, but likely limited the impact of the countermeasures. The only effects that a longer or larger study may clarify are the impact of countermeasures on VO_2_max and cardiac mass discussed above. Third, our study only included males and thus may not be generalizable to females undergoing spaceflight or bedrest. Finally, cardiac torsion and untwisting could not be assessed and thus our results are not directly comparable to prior studies that showed slowed untwisting after bedrest. We substituted it by circumferential strain and found no impact of countermeasures.

## Conclusion

During this 21-day HDBR RVE and NeX countermeasures did not limit losses in PV, OT and cardiac volumes. RVE and NeX might alleviate VO_2_max loss, and whey protein supplementation might be beneficial on myocardial mass but this would require further studies with larger sample size. Despite the reduction in left ventricle volumes during bedrest, deformation of the heart assessed by GCS was preserved and untwisting deformation was enhanced during orthostatic stress, illustrating the ability of the deconditioned heart to preserve systolic and diastolic functions.

## Data Availability Statement

The datasets generated for this study are available on request to the corresponding author.

## Ethics Statement

This study was reviewed and approved by French Health Authorities (Comité de Protection des Personnes Sud-Ouest Outre-Mer I). The subjects provided their written informed consent to participate in this study.

## Author Contributions

PG designed the study, acquired and analyzed the data, and wrote the manuscript. JM analyzed MRI data, wrote and reviewed the manuscript. MB acquired MRI data and reviewed the manuscript. FL performed and analyzed biochemical data and reviewed the manuscript. M-PB designed the study and acquired data. M-AC designed the study, acquired and analyzed the data and reviewed the manuscript. AP-L acquired data and reviewed the manuscript. BL designed the study, analyzed MRI data and reviewed the manuscript. NN designed the study, analyzed data, performed statistical analysis and reviewed the manuscript. All authors contributed to the article and approved the submitted version.

## Conflict of Interest

The authors declare that the research was conducted in the absence of any commercial or financial relationships that could be construed as a potential conflict of interest.
