# Supplementary figures and images for: MNX (Medium Duration Nutrition and Resistance-Vibration Exercise) Bed-Rest: Effect of Resistance Vibration Exercise Alone or Combined With Whey Protein Supplementation on Cardiovascular System in 21-Day Head-Down Bed Rest
Source: Front Physiol. 2020 Jul 16;11:812. doi: 10.3389/fphys.2020.00812 (PMC7378749; doi:10.3389/fphys.2020.00812)

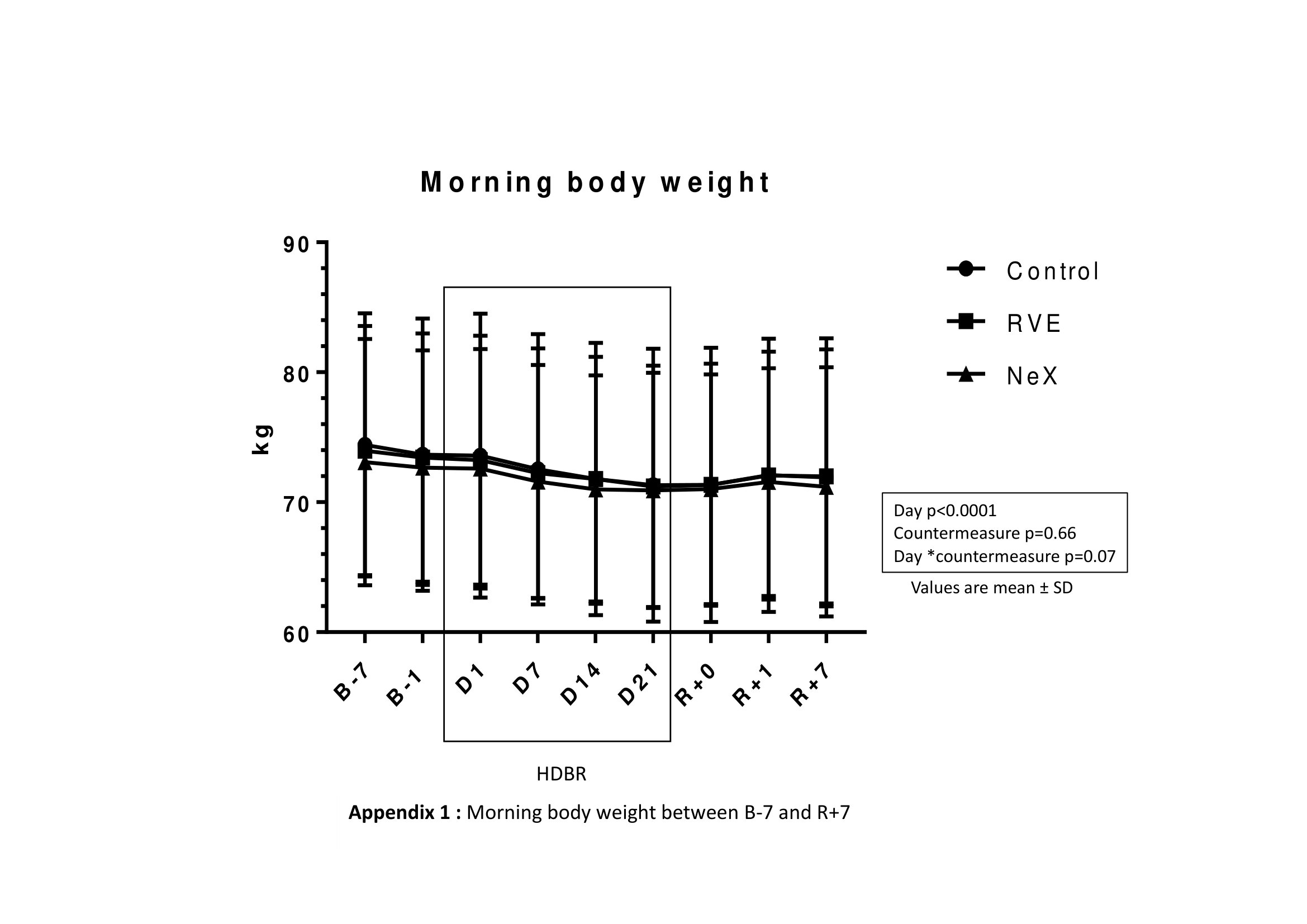

Supplement: Supplementary file 1 [file Image_1.JPEG]

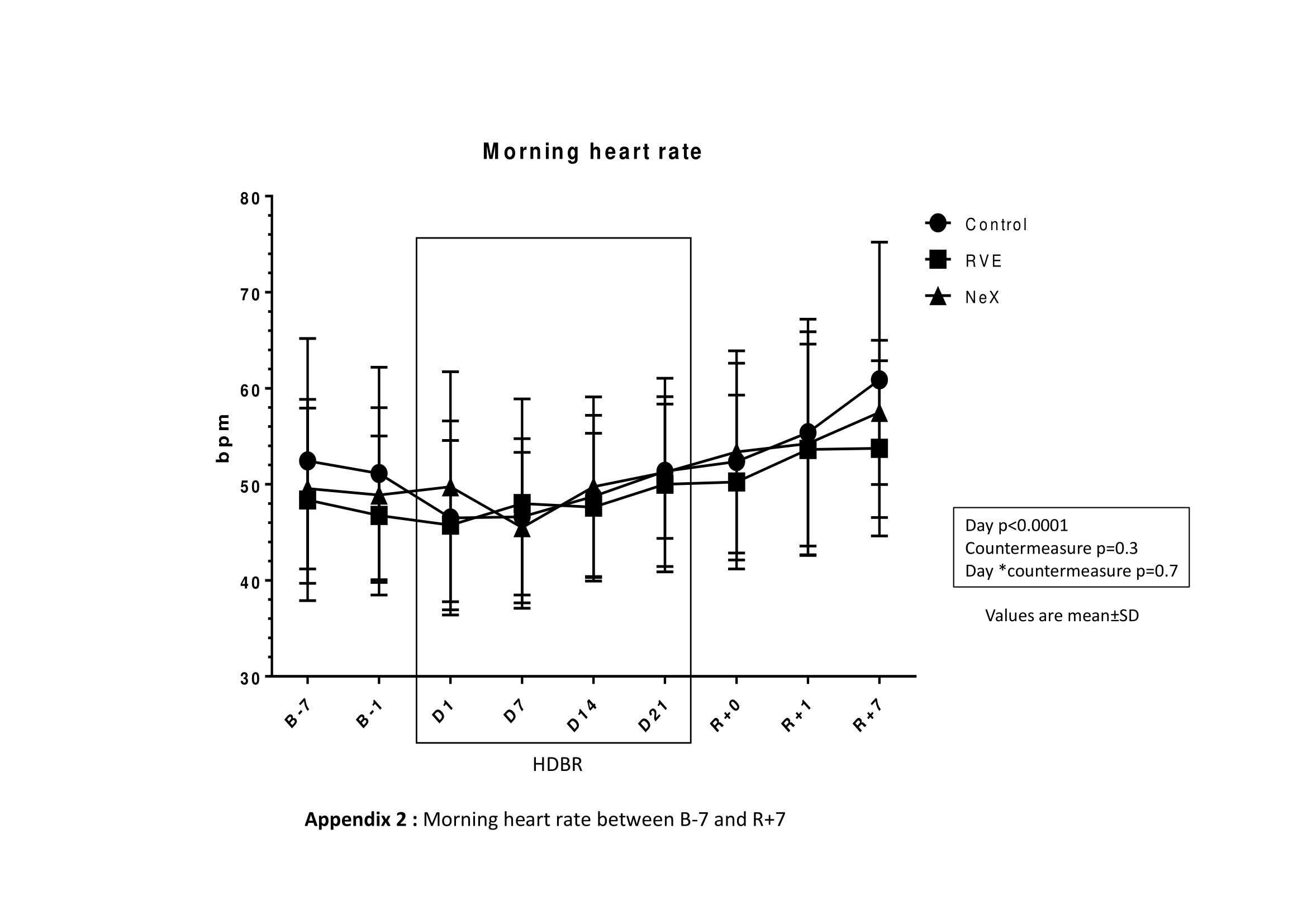

Supplement: Supplementary file 2 [file Image_2.JPEG]

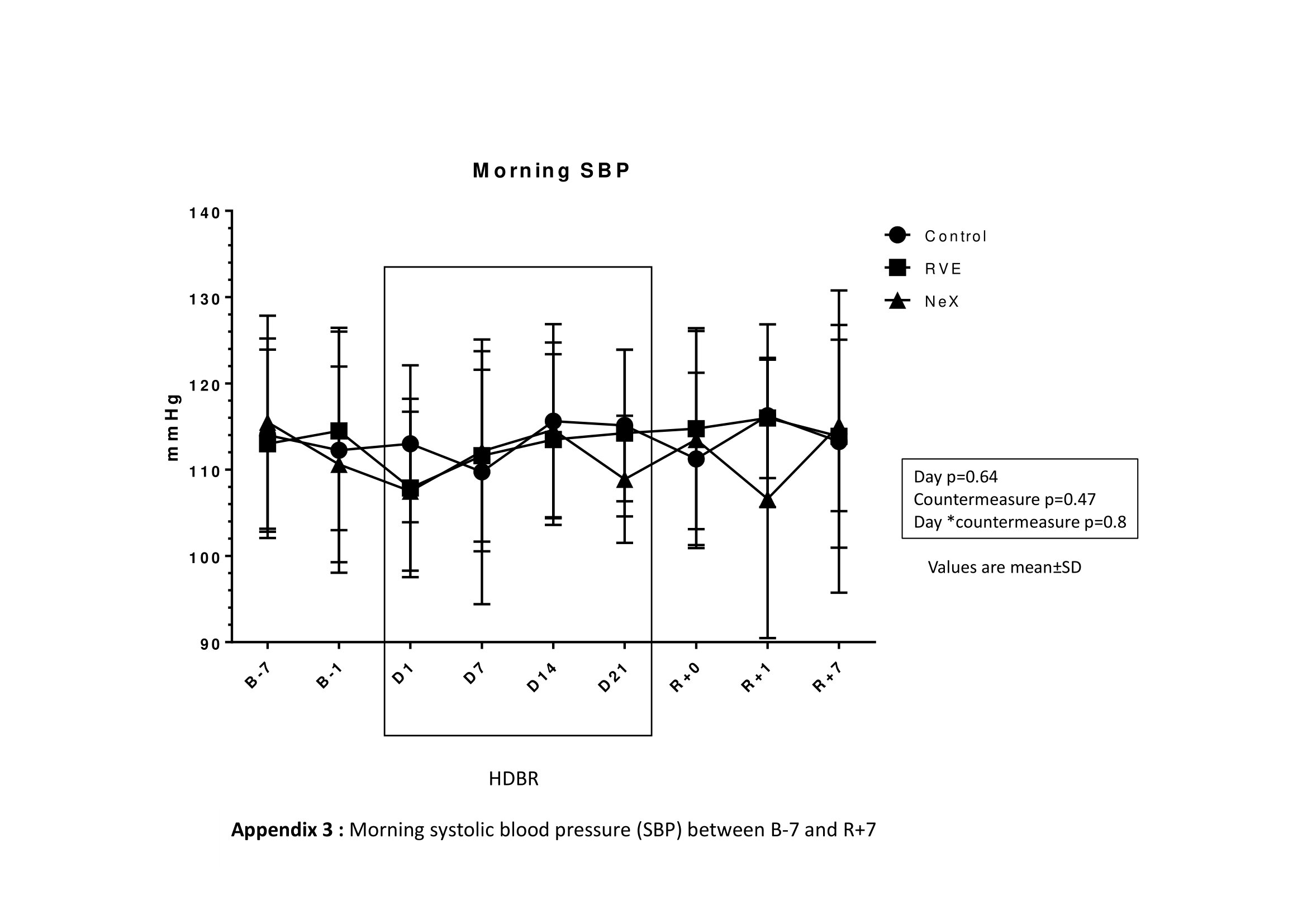

Supplement: Supplementary file 3 [file Image_3.JPEG]

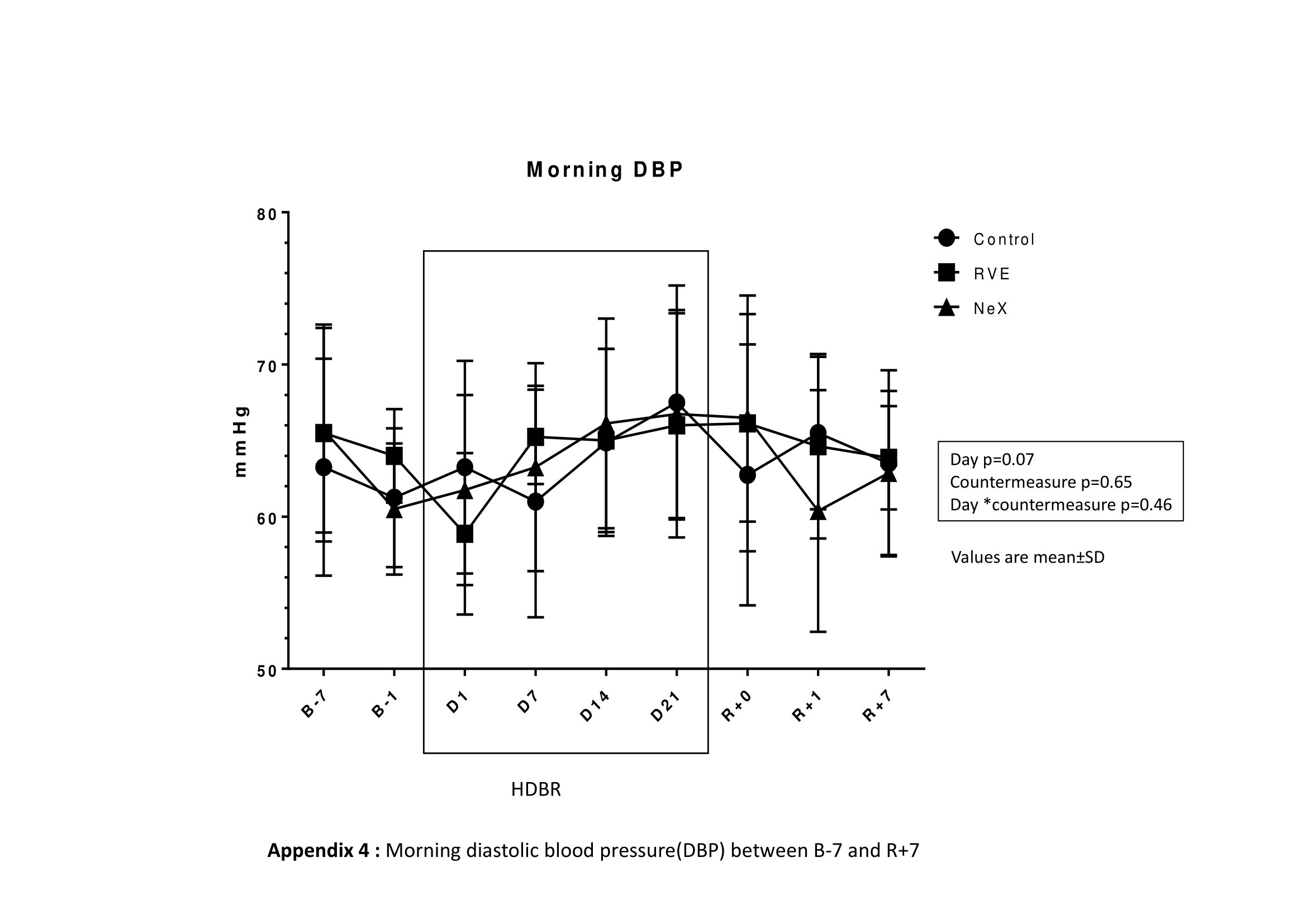

Supplement: Supplementary file 4 [file Image_4.JPEG]

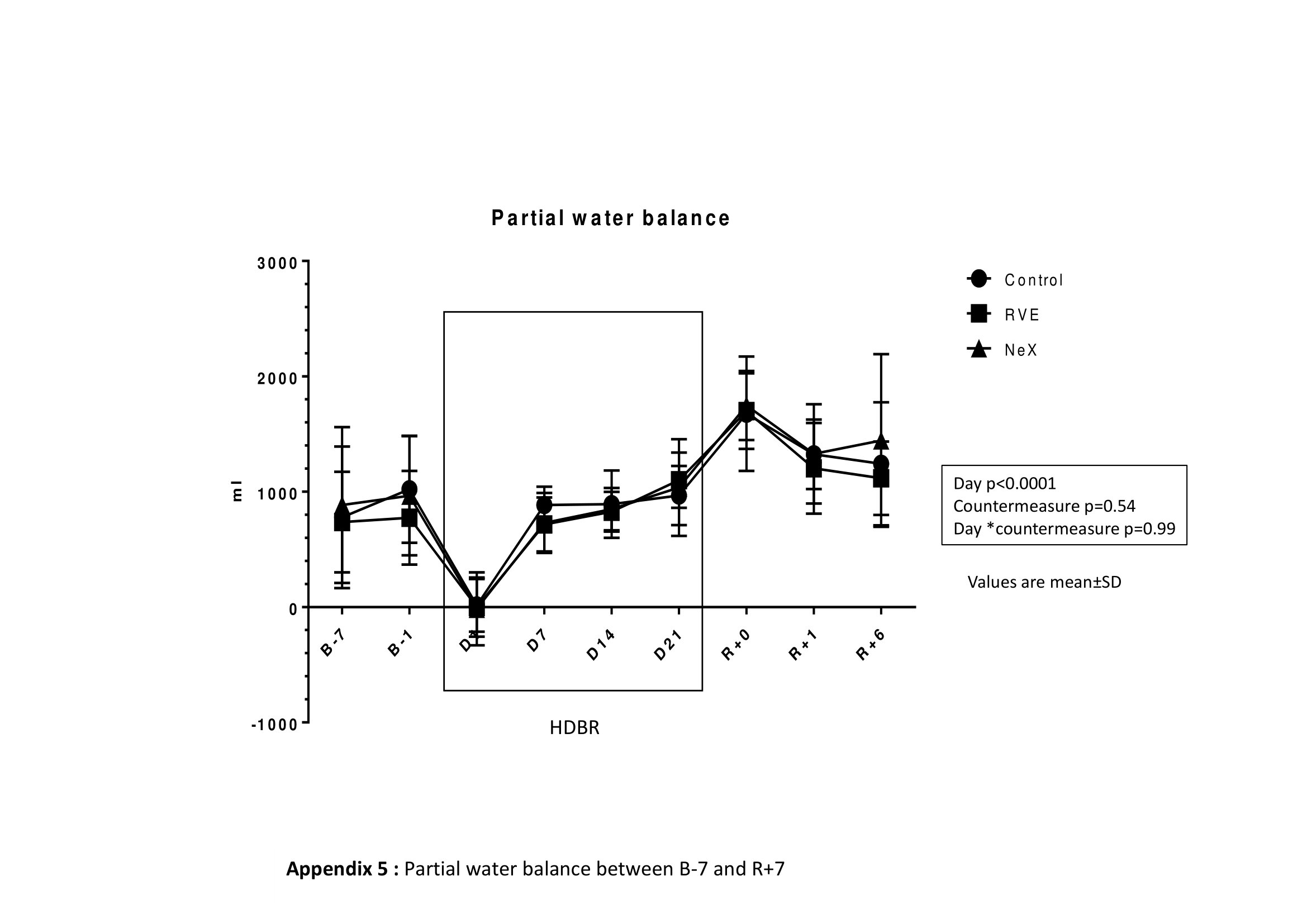

Supplement: Supplementary file 5 [file Image_5.JPEG]
